# Acetylcholine Inhibits Monomeric C-Reactive Protein Induced Inflammation, Endothelial Cell Adhesion, and Platelet Aggregation; A Potential Therapeutic?

**DOI:** 10.3389/fimmu.2018.02124

**Published:** 2018-09-26

**Authors:** Mark Slevin, Rocco S. Iemma, Yasmin Zeinolabediny, Donghui Liu, Glenn R. Ferris, Vittorio Caprio, Nicola Phillips, Mario Di Napoli, Baoqiang Guo, Xianwei Zeng, Raid AlBaradie, Naif K. Binsaleh, Garry McDowell, Wen-Hui Fang

**Affiliations:** ^1^Faculty of Science and Engineering, School of Healthcare Science, Manchester Metropolitan University, Manchester, United Kingdom; ^2^Institute of Dementia and Neurolgical Aging, Weifang Medical University, Weifang, China; ^3^University of Medicine and Pharmacy, Târgu Mures, Romania; ^4^Applied Medical Sciences College, Majmaah University, Al Majma'ah, Saudi Arabia; ^5^Neurological Service, Ospedale San Camillo de Lellis, Rieti, Italy

**Keywords:** CRP, inflammation, cell adhesion, acetylcholine, nicotine

## Abstract

**Objectives:** In this study, we examined the possibility of using targeted antibodies and the potential of small molecular therapeutics (acetylcholine, nicotine and tacrine) to block the pro-inflammatory and adhesion-related properties of monomeric C-reactive protein (mCRP).

**Methods:** We used three established models (platelet aggregation assay, endothelial leucocyte binding assay and monocyte inflammation via ELISA and Western blotting) to assess the potential of these therapeutics.

**Results:** The results of this study showed that monocyte induced inflammation (raised tumor necrosis factor-alpha-TNF-α) induced by mCRP was significantly blocked in the presence of acetylcholine and nicotine, whilst tacrine and targeted antibodies (clones 8C10 and 3H12) had less of or no significant effects. Western blotting confirmed the ability of acetylcholine to inhibit mCRP-induced cell signaling phosphorylation of extracellular signal regulated kinase 1/2 (ERK1/2), p38 and nuclear factor-kappa B (NF-κB). There was no evidence of direct binding between small molecules and mCRP. mCRP also induced endothelial cell-monocyte adhesion in a dose dependent fashion, however, both acetylcholine and nicotine as well as targeting antibodies notably inhibited adhesion. Finally, we investigated their effects on mCRP-induced platelet aggregation. All three small molecules significantly attenuated platelet aggregation as did the antibody 8C10, although 3H12 had a weaker effect.

**Discussion:** Acetylcholine and to a lesser extent nicotine show potential for therapeutic inhibition of mCRP-induced inflammation and cell and platelet adhesion. These results highlight the potential of targeted antibodies and small molecule therapeutics to inhibit the binding of mCRP by prevention of membrane interaction and subsequent activation of cellular cascade systems, which produce the pro-inflammatory effects associated with mCRP.

## Introduction

Activated platelets and endothelial leucocyte interactions represent an important link between pro-thrombotic and pro-inflammatory association of monomeric C-reactive protein (mCRP). These interactions may play a significant role in atherogenesis of cardiovascular diseases leading to acute ischemic stroke and a more chronic role in the development of brain lesions in vascular dementia and associated diseases (1).

It has been shown that cell membranes, liposomes, and static activated platelets can dissociate pentameric or native C-reactive protein (nCRP) into the monomeric and highly pro-inflammatory form via binding to phosphocholine groups on lysophosphatidylcholine. The main proposed mechanism of mCRP-associated interaction with cellular membranes and receptors is via its cholesterol binding domain (cystathionine-β-synthase; CBS; a.a 35–47). In this mechanism, the hydrophobic region of the mCRP inserts into lipid rafts of the plasma membrane binding to cholesterol molecules (2). Similarly, deposition of mCRP on to endothelial cell (EC)-circulating micro particles seems to be associated with chronic inflammation and linked to macrophage activation and T cell polarization (3).

Recently, Thiele et al. (4) described a phospholipase-A2 blocking mechanism using 2-(p-amylcinnamoyl)-amino-4-chlorobenzoic acid, (ONO-RS) that effectively prevented nCRP association with lysophosphatidylcholine on the cell surface and subsequent dissociation to mCRP concomitantly, significantly attenuating the pro-inflammatory effects of the protein both *in vitro* and *in vivo*. This work suggested that effective blocking of the binding of mCRP to the cell membrane could inhibit dissociation and abrogate the detrimental effects known to be associated with neurological inflammation and subsequent stroke worsening and/or dementia, as well as cardiovascular instability and complication (5). However currently considered anti-inflammatory molecules such as interleukin-1 receptor (IL-1R) antagonist may not be effective therapeutic agents being difficult to pass through the blood-brain-barrier (6) and/or having possible toxic and off pathway side-effects when given systemically at doses that could be useful therapeutically (7). Given the structural similarity between phosphocholine and acetylcholine we became interested in examining the potential of this neurotransmitter and two representative cholinergic small molecules, nicotine and tacrine, to perturb the actions of mCRP.

Here, we investigated the potential of a number of compounds anticipated to interact with mCRP/phosphatidylcholine in an effort to understand their capability and, subsequently, mechanism of action in blocking mCRP-mediated inflammation, EC-monocyte activation and platelet aggregation.

## Materials and methods

### Cell culture and differentiation

U937 cells were maintained in RPMI 1640 medium supplemented with 10% de-complemented Fetal Bovine Serum (FBS) under humidified 5% CO_2_ air at 37°C in a T-75 flask. The media was changed every 3 days. Cell viability was estimated using a Biorad TC1 automatic cell counter. Cell viability was maintained above 90% for the experiment. To induce monocyte differentiation into adherent macrophages, the U937 cells were seeded at an initial density of 2 × 10^6^ in 2 ml of differential media/well [growth media with phorbol-12-myristate 13-acetate (PMA) at 50 ng/ml for 72 h in a 6-well plate]. Following differentiation, the cells were washed twice with warm Dulbecco's Phosphate Buffered Saline (DPBS). Next, the macrophages were starved in RPMI 1640 medium supplemented with 2% FBS under humidified 5% CO_2_ air at 37°C for at least 24 h. Next, the macrophages were stimulated for 8 min with mCRP (for Western blotting based on our previously published observations) and 24 h for inflammation assays following 2 h pre-incubation with acetylcholine (10–100 μM), nicotine (0.93 μM) or tacrine (1 μM). Concentrations of small molecules were chosen based on published literature showing their use as inhibitors in macrophages/glia, [acetylcholine, (8)]; [nicotine, (9)]; [tacrine, (10)] and our own pilot observations and optimization (toxicity assay assessment of viability using a range of concentrations for the three molecules showed that the concentrations above were non-toxic to the U937 cells). For the monocyte-EC adhesion assay, immortalized human brain microvascular EC cells (HbMEC), were kindly donated by Prof. Babette Weksler (Division of Hematology and Medical Oncology, Weill Medical College, Cornell University, New York). Cells were cultured routinely before use in microvascular EC medium-2 (EBM-2) from Clonetics (Lonza, Germany), supplemented with growth factors as recommended by the manufacturer.

### ELISA assay

Human promonocytic leukemia U937 cells were grown in RPMI 1640 medium supplemented with 10% FBS, (Sigma-Aldrich) in a humidified incubator with 5% CO_2_ at 37°C. U937 monocytes (2 × 10^6^ cells/well) were fully differentiated into macrophages after 72 h incubation with 50 ng/ml phorbol-12-myristate 13-acetate (PMA) in 6-well culture plates. After washing twice with DPBS, macrophages were pre-treated with acetylcholine (10 μM), nicotine (0.93 μM), tacrine (5 μM), methyllycaconitine (10 μM), anti-CD16/32/64 (1:100), anti-mCRP antibody 3H12 clone (1:10), or anti-mCRP antibody 8C10 clone (1:10) for 2 h, followed by stimulation with mCRP (100 μg/mL) for an additional 24 h. Mouse monoclonal antibodies to human mCRP sub-unit (8C10/3H12) were obtained from Dr L.A. Potempa and fully characterized as described previously (11). We have previously shown that 8C10 (N-terminal aa-22-45) pre-incubation of EC was sufficient to block angiogenesis and associated cell signaling (12). Here, we employed the use of this antibody and a second similar one (3H12; C-terminal aa-198-206) as “potential” blocking antibodies in U937 inflammatory response.

The production of tumor necrosis factor alpha (TNF-α), IL-6, and IL-10 in the supernatant was quantified using ELISA kits (R&D Systems) according to the manufacturer's instruction. Stimulation with lipopolysaccharide (LPS) (10 ng/mL) for 24 h was used as the positive control for macrophage cytokine production. Samples were tested in triplicate and results are presented as the mean ± SD from a representative example of three independent experiments, unless specified otherwise in the text. ^*^P ≤ 0.05; ^**^P < 0.01; ^***^P < 0.001 using ANOVA.

### Western-blot protocol

A general RIPA buffer containing a protease and phosphatase inhibitor cocktail was used to make the cell lysates. Following this, the cell lysates were sonicated for 20 s and centrifuged for 10 min at 10000 RFC at 4°C. The supernatant protein samples were collected and the protein concentrations were estimated using the BCA protein assay. Then, the samples were frozen at −80°C for later use.

Equal quantities of proteins (30 μg) were mixed with 2× Laemmli sample buffer, boiled in a water bath for 15 min and then centrifuged. Samples were separated along with pre-stained molecular weight markers (32,000–200,000 kDa) by 12% SDS-PAGE. Proteins were electro-transferred (Hoefer, Bucks, UK) onto nitrocellulose filters (1 h) (Whatman, Protran BA85, Germany) and the filters were blocked for 1 h at room temperature in TBS-Tween (pH 7.4) containing 1% bovine serum albumin (BSA). Filters were then stained with the primary antibodies diluted in the blocking buffer, overnight at 4°C on a rotating shaker. The following primary antibodies were applied at 1:1,000 dilution: phospho/total-extracellular signal-regulated kinase 1/2 (ERK1/2) (thr202-tyr204; mab/4370 and mab/4695, respectively; from Cell Signaling Antibodies, Bio-rad, Hertfordshire, UK); phospho/total-jun N-terminal kinase 1/2 (JNK1/2) (t183, y185, mab/1205 and mab ab179461, respectively; from Bio-Techne Ltd., Minneapolis, USA); phospho/total-p38 (t180, y182 ab4822, and ab27986, respectively; from Abcam, West Sussex, UK); and phospho/total-nuclear factor kappa B (NFκb) (p65, S529 and p65, ab16502; from Abcam, West Sussex, UK).

After washing (5 × 10 min in TBS-Tween at room temperature), filters were stained with either goat anti-rabbit or rabbit anti-mouse HRP-conjugated secondary antibodies diluted in TBS-Tween containing 5% de-fatted milk (1:2,000, 1 h, room temperature) with continuous mixing. After a further five washes in TBS-tween, proteins were visualized using enhanced chemiluminescence detection (ECL, Thermo Scientific, UK), and semi-quantitatively identified fold differences compared with house-keeping controls (α-tubulin, ab7291, Abcam, West Sussex, UK) were determined using Image-Lab software (Bio-rad, UK). All experiments were repeated three times and a representative example is shown.

#### NF-κB translocation assay

Macrophages were cultured alone or in the presence of LPS (10 μg/ml) as a positive control or mCRP (100 μg/ml) with and without small molecules (2 h pre-incubation as described above) on glass coverslips for 1 h prior to a 5 min wash in PBS. Samples were fixed in 100% methanol at−20°C for 5 min and following evaporation, stored at −80°C.

Prior to staining, cells were rehydrated with 0.05% PBST. Non-specific binding of the secondary antibody was blocked using 4% goat serum (Vector laboratories, Peterborough, UK) for 30 min. Cells were washed with PBST (2 × 5 min) then incubated overnight with NF-κB p65 rabbit mAb 16502 (Cell Signaling, MA, USA) at 1:400 dilution. Cells were then rinsed twice with PBST for 5 min, and incubated with goat anti-rabbit IgG secondary antibody (Alexa Fluor 488; Thermo-Fisher scientific, Runcorn, UK) at 1:250 dilution. Slides were washed with PBS for 5 min, mounted with vector shield (H1200 with DAPI) and left to dry for 20 min before microscopy. Fluorescence images were captured on a Zeiss Z1 AxioObserver fluorescence microscope. Three coverslips/wells were used for each test and 500 cells were counted from each coverslip; and the experiment performed twice. Differences in relative translocation were analyzed using one-way ANOVA with Bonferroni post-test analysis.

### Cytoselect monocyte-endothelium adhesion assay

This was applied according to the manufacturer instruction and referring to the work of Kapitsinou et al. (13). Briefly, human brain microvessel EC (HbMEC) (1 × 10^5^/per well) were added applied to a 96-well plate. After 48 h, when the EC monolayer was formed, they were treated with mCRP (1–100 μg/ml) for 6 h. After removing the medium, they were washed once with serum free medium, and 200 μl of the monocyte suspension already labeled with Leuko-Tracker added to each well and incubated for 90 min. After removing the medium and a further three washes, 150 μl of 1X lysis buffer was added to each well containing cells. Fluorescence was measured using a fluorescence plate reader at 480 nm/520 nm. In these experiments we added nCRP since possible effects on EC-monocyte interactions have not previously been examined. However since published data clearly shows a lack of inflammatory activity on macrophages (3, 14), we did not include it within the other experimental protocols. Each test was conducted in triplicate, repeated three times, and a representative example is given. ^*^p ≤ 0.05 using Wilcoxon matched pair test.

### Platelet aggregation assay

Venous blood was taken from non-smoking (since smoking is known to affect/increase platelet aggregation) (15), healthy volunteers with informed consent (carried out with internal ethical approval obtained through our local University ethical committee). Monomeric C-reactive protein-induced platelet activation was evaluated using the platelet aggregation assay, and its coagulation was measured by light-transmission aggregometry (LTA) using platelet rich plasma (PRP). Blood was centrifuged (20 min, 150 g, 20°C) to obtain PRP.

A total of 250 μL of PRP adjusted to 250 × 10^6^ platelets/mL was incubated with 250 μL solution containing mCRP (100 μg/ml) or control buffer and small molecules. Adenosine diphosphate (ADP) at 10 μM was used as a positive control. Antibodies were used at 1:100 dilution. The following concentrations were used for the small molecules, mCRP 100 μg/mL + nicotine 0.93 μM, mCRP 100 μg/mL + acetylcholine 10 μM and mCRP 100 μg/mL + tacrine at 10 μM. Each experiment was performed in triplicate. Results are presented as the mean ± SD of maximum platelet aggregation (%), from a representative example of three independent experiments. ^*^P ≤ 0.05; ^**^P < 0.01; ^***^P < 0.001 using ANOVA.

#### CRP purity testing

In all of the experiments CRP treated with detoxi-gel columns (CRPdt) containing immobilized polymyxin B was used, to ensure the absence of pyrogens/endotoxin (AffinityPak™ detoxi-Gel™ column; Pierce, Rockford, IL) and removal was confirmed using the Limulus assay. Sources were purchased free from sodium azide.

### Dot-blot assay

#### Incubation of mCRP with small molecules

mCRP was incubated for 2 h with tacrine (10 μM), nicotine (0.93 μM) or acetylcholine (100 μM) prior to binding assessment on nitrocellulose described below.

#### Dissociation of nCRP to mCRP

The nCRP commercial sample was purchased from Yo protein laboratories (Aachen, Germany). 250 μL of mCRP was added 250 μL of 10 mM EDTA and 8 M urea for chelation and the mixture incubated at 37°C for 2 h, with or without tacrine (10 μM), nicotine (0.93 μM), or acetylcholine (100 μM).

A grid was drawn on a piece of nitrocellulose membrane using a pencil and indicating the region for the blots. Next, using a narrow-mouth pipette tip, 2 μl of samples was placed onto the nitrocellulose membrane at the center of the grid. The penetration area was kept to a minimum by applying it slowly. After drying, non-specific sites were blocked with 5% BSA in TBS-T (0.5–1 h, RT). A 10 cm petri dish was used as a reaction chamber, and samples were incubated with primary anti-mCRP/nCRP antibodies [1:10] dissolved in BSA/TBS-T for 30 min at RT. After washing three times with TBS-T (3 × 5 min), samples were incubated with secondary anti-mouse antibody conjugated with HRP (1:500 for 30 min at RT).

After washing, (TBS-T; 15 min × 1 and 5 min × 2), then once with TBS (5 min), membranes were incubated with ECL reagent for 1 min, covered with Saran-wrap (after removal of excessive solution from the surface), and exposed using the G-box/Image Lab software. All blots were performed in triplicate and experiments were repeated three times with a representative example being shown.

### Surface plasmon resonance testing

Surface plasmon resonance (SPR) was used to assess the binding of mCRP to nicotine, acetylcholine, tacrine and 3H12 polyclonal antibody. Monomeric CRP was first buffer exchanged using a 0.5 ml Zeba^TM^ spin column (Thermo) which was pre-equilibrated in PBS. mCRP was then incubated with a 1:2 ratio of NHS-Peg4-Biotin (Thermo) for 30 min at room temperature before purifying the free biotin with another Zeba^TM^ spin column in PBS. SPR was performed on a Biacore T200 (GE life sciences) and a streptavidin coated SA chip. The instrument was equilibrated in Biacore buffer, 10 mM HEPES buffer pH 7.4 with 0.05% tween 20 and the SA chip was washed with 10 mM EDTA and 50 mM NaOH prior to loading with biotinylated mCRP. For the antibody binding test the chip was loaded with ~100 response units of mCRP. For the small molecule tests the chip was loaded to a maximum loading of ~3,500 response units of biotinylated mCRP. Analytes, either antibody at 1:100 dilution, 1 mM nicotine, 1 mM acetylcholine, or 10 μM tacrine diluted in Biacore buffer were injected at 30 μl/min over the mCRP surface and the response monitored in real time. The results are shown as a subtracted response where flow cell 1 was used as a reference with no mCRP added which was subsequently subtracted from the data.

### Statistical analyses

Data are presented as the mean ± SD of individual representative experiments carried out in triplicate. Statistical analysis was performed using GraphPad Prism software version 7.0 for Windows (GraphPad Software). The values were compared using paired Student's *t*-test, non-parametric Wilcoxon test, or one-way ANOVA with Bonferroni post-test analysis. ^*^P ≤ 0.05; ^**^P < 0.01; ^***^P < 0.001.

## Results

### mCRP modulated TNF-α, IL-6, and IL-10 expression in U937-derived macrophages

**Figure 1:**
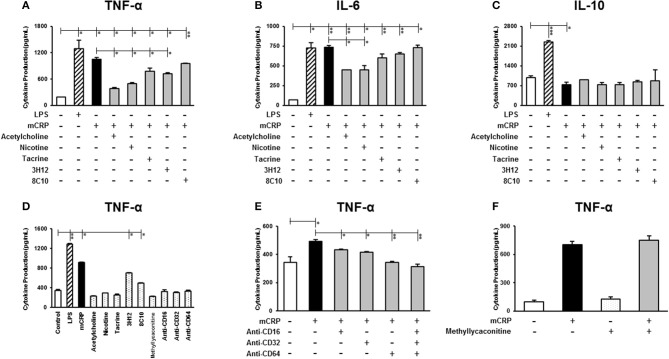
The effect of mCRP on macrophage cytokine production. The differentiated U937 macrophages were pre-treated with acetylcholine (10 μM), nicotine (0.93 μM), tacrine (5 μM), anti-mCRP antibodies 3H12 (1:10) or 8C10 (1:10) for 2 h, followed by stimulated with mCRP (100 μg/mL) for an additional 24 h. The production of TNF-α **(A)**, IL-6 **(B)**, and IL-10 **(C)** in the supernatant was quantified using ELISA kits (R&D System). The stimulation with LPS (10 ng/mL) for 24 h was used as the positive control for macrophage cytokine production. **(D)** Shows that treatment with small molecules alone, nicotinic acid receptor antagonist methyllycaconitine (10 μM) or CD16/32/64 to FC-ɤ receptors (1:100) did not affect cytokine (TNF-α) production in U937 cells, although there was a marginal increase in TNF-α in the presence of the mCRP antibodies probably due to residual contamination of low levels of LPS. When cells were pre-treated with CD16/32/64 to FC-ɤ receptors (1:100) for 2 h, complete abrogation of mCRP-induced TNF-α production was shown **(E)**, whilst pre-incubation with the nicotinic acid receptor antagonist methyllycaconitine (10 μM) for 2 h in the presence of either acetylcholine or nicotine did not reverse the anti-inflammatory capacity **(F)**. Results are presented as the mean ± SD from a representative example of three independent experiments. ^*^P ≤ 0.05; ^**^P < 0.01; ^***^P < 0.001 using one-way ANOVA with Bonferroni post-test analysis.

To determine the effect of mCRP on the expression of inflammatory mediators, U937-derived macrophages were exposed for 24 h to 100 μg/ml mCRP and secreted protein levels of TNF-α, IL-6, and IL-10 in the supernatant were quantified using ELISA. As shown in Figure 1, mCRP significantly increased the secretion of pro-inflammatory cytokines including TNF-α (*P* < 0.05, Figure 1A) and IL-6 (*P* < 0.01, Figure 1B), exerting a similar response to LPS. Surprisingly, mCRP significantly decreased the levels of anti-inflammatory cytokine IL-10 by 25% (derived from our included representative experiment and similarly decreased in repeated tests; *P* < 0.05, Figure 1C), opposite to LPS which augmented the production of IL-10 by 2.2-fold (*P* < 0.001, Figure 1C).

We then investigated whether the small molecules (acetylcholine, nicotine, tacrine) and anti-mCRP antibodies (3H12 and 8C10) would affect macrophage cytokine profiles induced by mCRP. Pre-treatment with acetylcholine, nicotine, tacrine and anti-mCRP antibody 3H12 significantly inhibited TNF-α production induced by mCRP, with the strongest inhibition by acetylcholine (63.8% reduction), followed by nicotine (52.6% reduction), 3H12 (31.8% reduction), and tacrine (25.9% reduction) (derived from our included representative experiment and similarly decreased in repeated tests; ANOVA, *P* < 0.05, Figure 1A). Both acetylcholine and nicotine also significantly decreased IL-6 levels induced by mCRP (*P* < 0.05, Figure 1B). In addition, acetylcholine and 3H12 tended to restore the mCRP repressed IL-10 levels back to normal (*P* < 0.05, Figure 1C). Small molecules alone did not significantly increase macrophage cytokine expression (values for TNF-α shown in Figure 1D). A small increase in TNF-α was seen in the presence of 3H12/8C10 alone (in the absence of mCRP) and this was probably due to a residual low concentration of endotoxin found in the supernatant. To confirm receptor interaction of mCRP on the cell surface of macrophages we pre-incubated U937 with a pharmacological antagonist of nicotinic α7 receptor (methyllycaconitine; 10 μM) or blocking antibodies CD16/32/64 to FC-ɤ receptors (1:100) for 2 h. A combination of antibodies CD16/32/64 was able to completely abrogate mCRP-induced TNF-α production (*P* < 0.01, Figure 1E), whilst pre-incubation with the nicotinic receptor antagonist methyllycaconitine (10 μM shown and 1–100 μM tested) for 2 h in the presence of nicotine did not reverse the anti-inflammatory effects suggesting a pathway not working through the nicotinic receptor signaling (Figure 1F). Acetylcholine, nicotine and methyllycaconitine cytotoxicity studies are included as Supplementary Figure 1, and no cytotoxicity was observed at any of the concentrations used.

#### mCRP induced the phosphorylation of MAPK and activation of NF-κB in cultured macrophages

**Figure 2:**
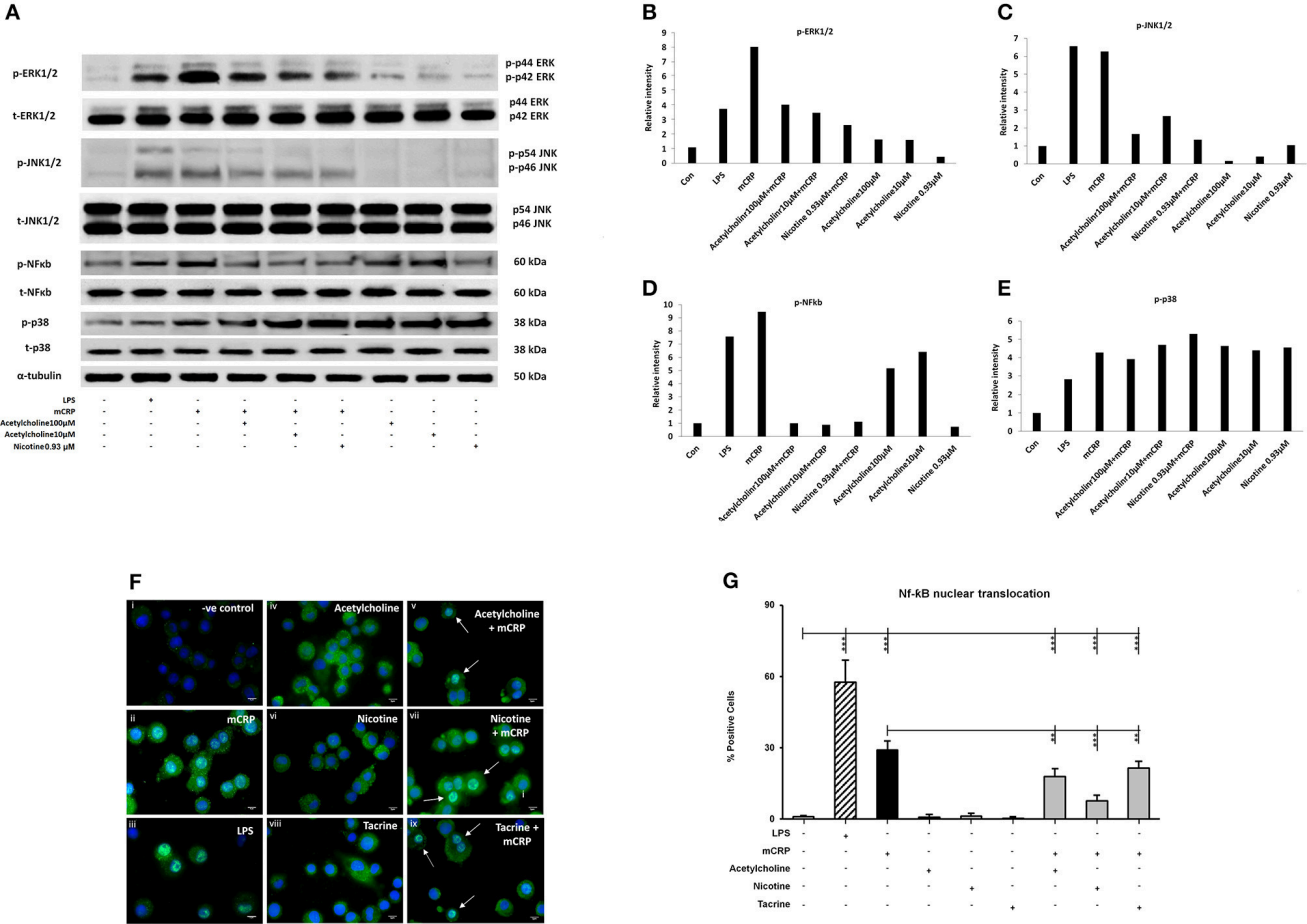
The expression of selected phospho-proteins by macrophages. 2 × 10^6^ (U937 cells) were differentiated in one well of a 6-well plate. After 72 h, when the macrophage monolayer had formed, they were treated with various concentrations of acetylcholine (10, 100 μM) or nicotine (0.93 μM) with or without mCRP (100 μg/ml) for 8 min. After removing the medium, they were washed twice with ice-cold PBS, followed by addition of 200 μl of 1X lysis Buffer to each well. Lane 1, control; Lane 2, LPS; Lane 3, mCRP; Lane 4, acetylcholine (10 μM) + mCRP; Lane 5, acetylcholine (100 μM) + mCRP; Lane 6, nicotine + mCRP; Lane 7, acetylcholine alone (100 μM); Lane 8, acetylcholine alone (10 μM); Lane 9, nicotine alone. (**A)** Expressions of selected proteins. **(B)** Bar chart shows phospho-ERK1/2 expression. **(C)** Bar chart shows phospho-JNK expression. **(D)** Bar chart shows phospho-p38 expression. **(E)** Bar chart shows phospho-NFκb expression. **(F)** Shows immunofluorescent demonstration of nuclear translocation of NFκb FITC green with DAB-blue positively stained nuclei (I, negative control; ii, mCRP treated for 1 h; iii, LPS positive control; iv, acetylcholine; v, acetylcholine + mCRP; vi, nicotine; vii, nicotine + mCRP; viii, tacrine; ix, tacrine + mCRP), and **(G)** is a representative bar chart showing the percentage of cells showing nuclear translocation from 500 counted cells/coverslip. Pre-incubation with nicotine (0.93 μM; 1 h) significantly reduced NFκb translocation. Each test was carried out in triplicate and the experiments conducted twice. A representative example is given here. ^**^P < 0.01; ^***^P < 0.001 using one-way ANOVA with Bonferroni post-test analysis.

**D**ifferentiated U937 macrophages were pre-treated with acetylcholine (100 μM, lane 4; 10 μM, lane 5) or nicotine (0.93 μM, lane 6) for 2 h, followed by stimulation with mCRP (100 μg/mL, lanes 3–6) for an additional 8 min (Figure 2A). Since tacrine was not effective in blocking macrophage-induced inflammation (Figure 1) we did not include it in these signaling experiments. The first lane was loaded with the extract from untreated macrophages as control. The cultured macrophages treated with LPS (10 ng/mL) for 8 min were used as a positive control (lane 2). Results show that acetylcholine was effective in blocking phosphorylation of ERK1/2, NF-κB, and JNK1/2 but not p-38 MAP kinase. Nicotine similarly inhibited phosphorylation of ERK1/2 JNK1/2 and NF-κB but not p38 signaling molecules. Figures 2B–E shows densitometric analysis of immunoblot quantification. Interestingly, acetylcholine alone increased NF-κB phosphorylation as well as that of p-38 MAP kinase, whilst increased p-38 phosphorylation was also seen after addition of nicotine. No change in the expression of “total” proteins was seen.

To confirm activation of NF-κB, we performed immunofluorescent staining on cultured U937 macrophages exposed to mCRP (100 μg/ml; 1 h) or LPS as a positive control (10 ng/ml). Nuclear translocation of NF-κB was clearly seen in LPS-treated cells (~63% of cells; iii) and also mCRP treated U937 (~30%; ii) but not in control untreated cells (< 1%; i) as shown in Figures 2F,G (*P* < 0.05 from a representative experiment, which was repeated giving similar results). None of the small molecules alone had any effect on translocation (iv, vi, and viii), however pre-incubation of cells with nicotine (0.93 μM; 1 h) significantly reduced the translocation of NF-κB (*p* < 0.05; from 30 to 18% in our presented experiment which was performed twice giving similar results). This data confirmed mCRP-induced activation of the NF-κB signaling pathway with gene transcriptional involvement.

#### mCRP-induced EC-monocyte adhesion tends to be inhibited by 8C10 antibody

**Figure 3:**
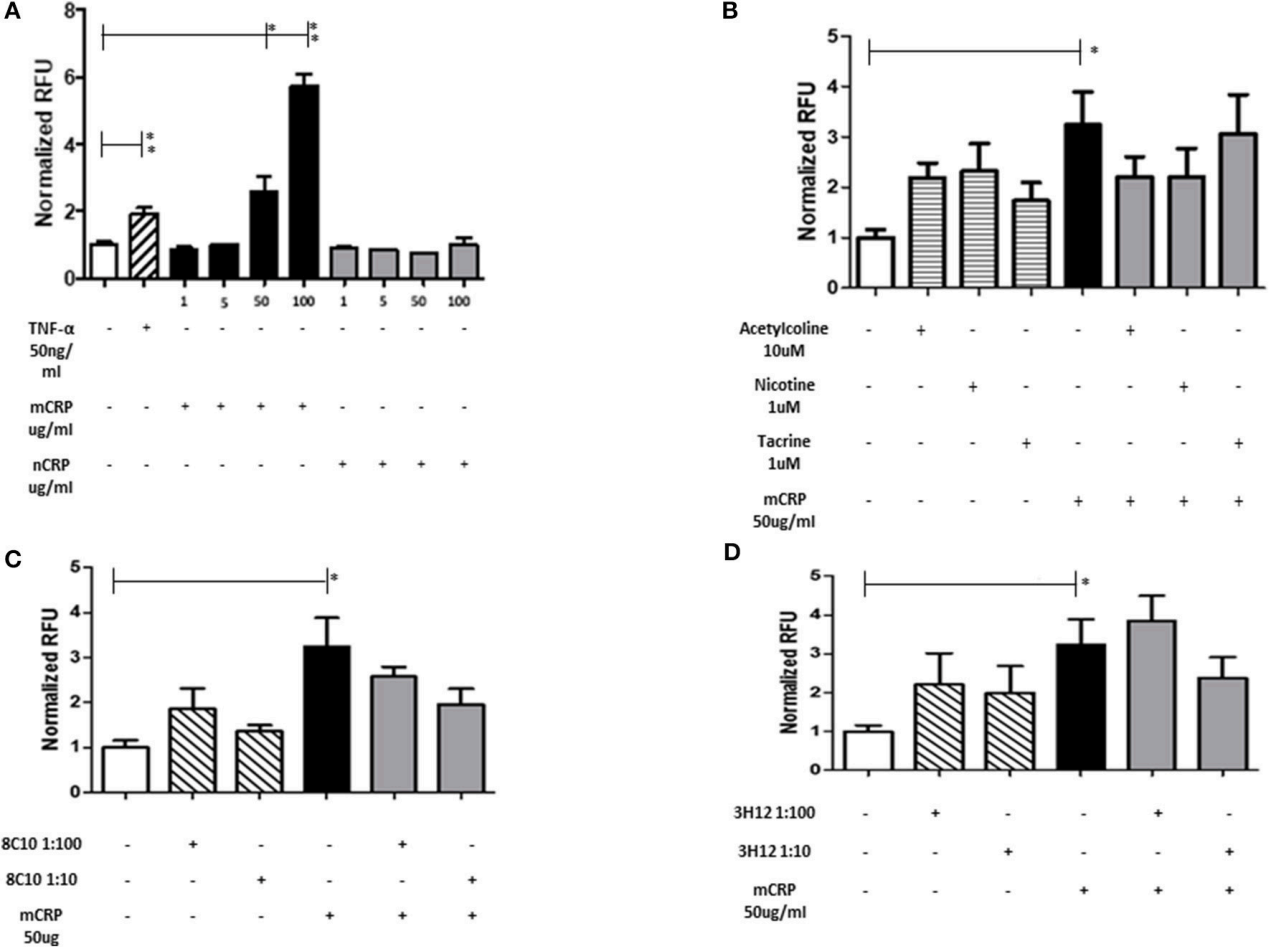
The effect of mCRP on endothelial cell adhesion to U937. After treating with mCRP for 6 h (10–100 μg/ml), monolayer HBmEC were incubated with U937 cells labeled with Leuko-Tracker for 60 min. Fluorescence of the control wells containing EC and U937 without any treatment were arbitrarily set as 1. **(A)** mCRP significantly increased EC adhesion to U937 in a dose dependent manner, however nCRP had no significant effect. **(B)** Acetylcholine (10 μM), and nicotine (0.93 μM), alone significantly promoted EC adhesion to U937, however, in the presence of mCRP, they antagonized mCRP-mediated adhesion of EC to U937 (non-significant). **(C)** Monomeric C-reactive protein specific antibody 8C10 also inhibited mCRP-mediated EC adhesion to U937. **(D)** mCRP specific antibody 3H12 reduced the aggregation but to a lesser extent than 8C10 (non-significant). Each test was conducted in triplicate, repeated three times, and a representative example is given. ^*^p ≤ 0.05, ^**^p < 0.01 using Wilcoxon matched pair test. Using ANOVA, no statistical differences were found in the inhibition of mCRP-driven EC adhesion to U937 cells.

Monocyte adherence to the endothelium is a strong indicator of abnormal activity/activation and potential for inflammatory signaling associated with vascular damage that may ultimately lead to atherosclerosis. mCRP significantly increased EC adhesion to monocytes in a dose dependent manner whilst native CRP (nCRP) had no significant effect (Figure 3A). Acetylcholine and nicotine alone significantly promoted EC adhesion to monocytes (*P* < 0.05), however, in the presence of mCRP, they tended to antagonize mCRP-mediated adhesion of EC to monocytes (non-significant; Figure 3B). mCRP-induced EC-monocyte adhesion tended to be inhibited by 8C10 particularly at 1:10 dilution (Figure 3C). Figure 3D shows that mCRP specific antibody 3H12 had a weak but non-significant inhibitory effect at 1:10 dilution only. Each test was conducted in triplicate and statistical analysis performed using the Wilcoxon matched pair test. Experiments were repeated three times and a representative example is given.

#### Small molecules and antibodies effectively blocked platelet aggregation

**Figure 4:**
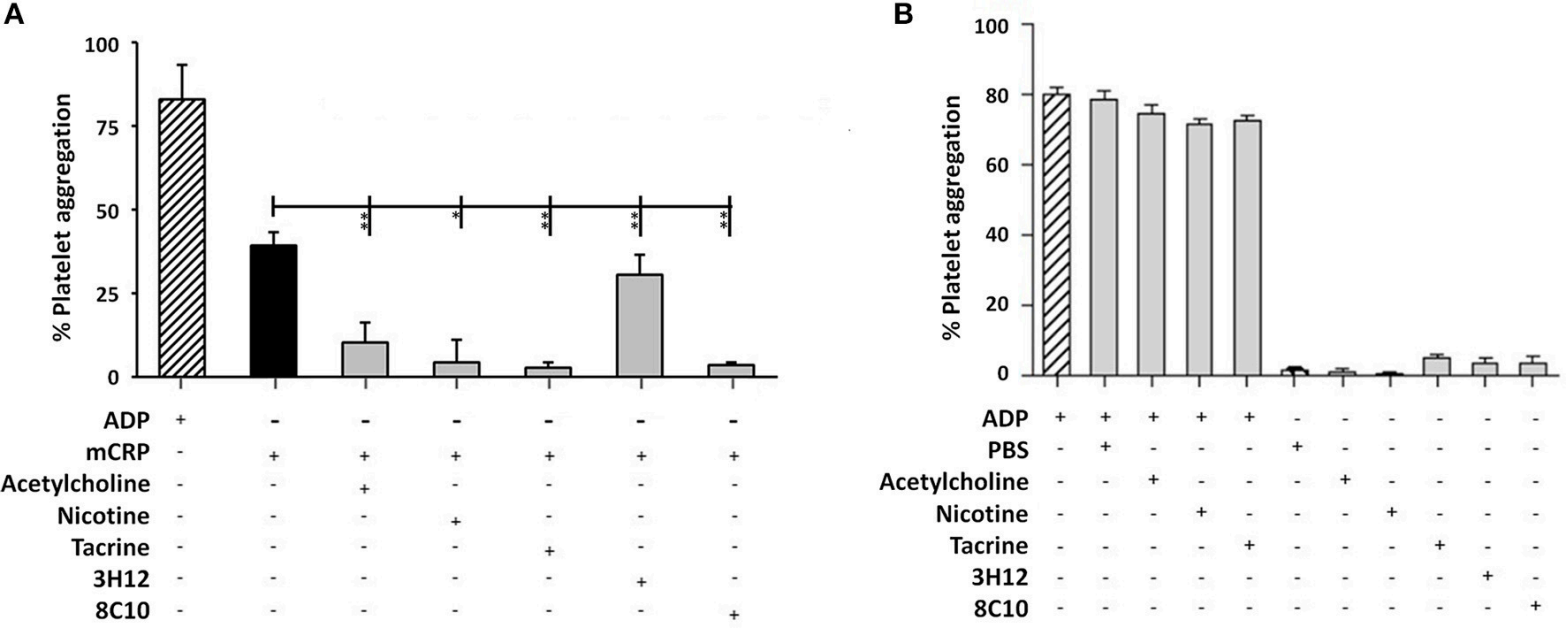
Platelet aggregation assay: **(A)** Platelet aggregation/ coagulation was measured by light-transmission aggregometry (LTA) using PRP derived from volunteers fresh whole blood. Monomeric C-reactive protein (100 μg/ml)-induced platelet aggregation was blocked by anti-mCRP antibody (8C10) and partially inhibited by anti-mCRP (3H12). Acetylcholine (10 μM), nicotine (0.93 μM), and tacrine (5 μM), almost completely inhibited mCRP-induced platelet aggregation (*P* < 0.05), whilst having no effect either alone or on ADP-induced platelet aggregation **(B)**. The experiments were each performed in triplicate. Results are presented as the mean ± SD from a representative example of three independent experiments. ^*^P ≤ 0.05; ^**^P < 0.01; using one-way ANOVA with Bonferroni post-test analysis.

The measurement of platelet aggregation is a strong indicator of the potential for thrombus or clot formation in acute coronary syndromes. Platelet aggregation analysis revealed that mCRP (100 μg/ml; 5 min), induced platelet aggregation (40–50%), as seen in Figure 4A. Monomeric C-reactive protein-induced platelet aggregation was blocked in the presence of the anti-mCRP antibody (8C10), and partially prevented by anti-mCRP antibody (3H12). All three small molecules almost completely inhibited mCRP-induced platelet aggregation (*P* < 0.05), but did not block aggregation induced by ADP (10 μM; Figure 4B). Monomeric C-reactive protein-induced aggregation of platelets was independently tested using a second healthy donor and similar levels of aggregation were produced (Supplementary Figure 2).

### Antibody and protein binding studies indicated no direct interaction between small molecules and mCRP

**Figure 5:**
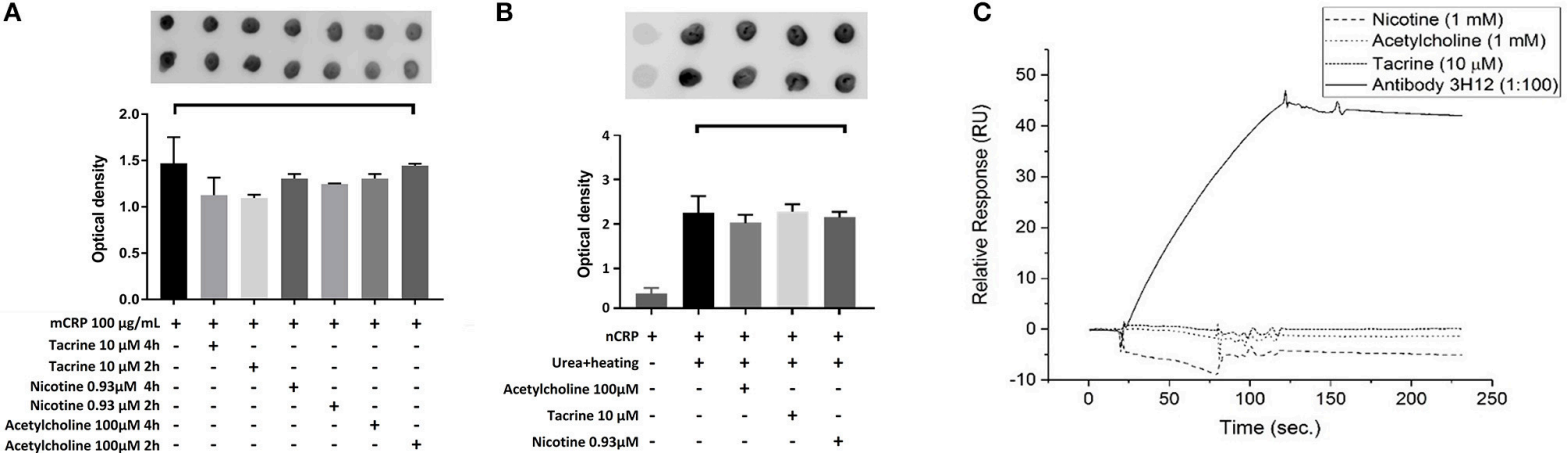
Dot-blot and cell binding assays: **(A)** After Incubation of mCRP with and without tacrine, nicotine, and acetylcholine for 4 h, dot blotting with 3H12 showed no significant specific antibody binding. **(B)** Following incubation of nCRP with urea at 37°C dot blotting using 3H12 to bind to the mCRP indicated that dissociation of nCRP to mCRP was not affected by the presence of any of the three small molecules. **(C)** The results of SPR showed that there was no observable interaction of the small molecules to biotinylated mCRP. The 3H12 antibody exhibited a strong response with a slow off-rate (kd) of ~7.8 × 10^−4^ s^−1^. Experiments were performed in duplicate and repeated three times. Results are presented as the mean ± SD from a representative example. No significant differences were found using one-way ANOVA with Bonferroni post-test analysis.

Here, we used dot blotting with our specific mCRP antibody 3H12 in order to examine possible interactions that would affect surface structure change/binding. No significant change in specific antibody binding was found between the mCRP control sample and mCRP samples pre-treated for either 2 or 4 h with any of the three small molecules (Figure 5A). After incubation of nCRP with urea at 37°C, dot blots obtained following incubation with mCRP antibody 3H12 indicated that urea mediated dissociation of nCRP to mCRP was not affected by acetylcholine, tacrine or nicotine (Figure 5B).

SPR binding analysis was used to identify any adsorption of material on to the mCRP-coated electrodes. Results indicated that under these conditions there was no observable direct interaction of any of the small molecules to biotinylated mCRP. The 3H12 antibody, however, exhibited a strong response with a slow off-rate (kd) of ~7.8 × 10^−4^ s^−1^ (Figure 5C). For each experiment, duplicates of each sample were made, and three repeats of each experiment were carried out with a representative example being shown here.

## Discussion

mCRP is involved in significant perpetuation of tissue associated inflammation, potentially associating it to thrombosis in atherosclerosis (14), neurological degradation and dementia (16), macular degeneration (17), and sepsis (18). Therefore, antagonists of either, native CRP breakdown and dissociation into mCRP, or small molecules that would inhibit mCRP binding and activation via the cell membrane phosphocholine docking site, could prove useful as future therapeutic agents.

Apart from our characterized antibodies (3H12 and 8C10; 8C10 having been previously shown by ourselves to block mCRP-induced cell signaling through p-ERK1/2 and angiogenesis in bovine aortic EC); (16), we investigated the potential of acetylcholine, nicotine and tacrine to modulate mCRP-induced inflammation. Acetylcholine is very similar in structure to phosphocholine. Work by Nazarov et al. (19) indicates that CRP could bind to acetylcholine, as evidenced, by CRP-based inhibition of breakdown of this molecule and subsequent influence on cardiovascular systemic inflammation. In order to gain some insight into potential CRP/acetylcholine interactions and how this compares to binding at known cholinergic receptors/enzymes; we also chose to examine the effects of nicotine – the putative agonist at nicotinic receptors and tacrine, a therapeutically useful inhibitor of acetylcholinesterase.

The induction of inflammation in U937-derived macrophages *in vitro* is a reliable indication of the activity of mCRP, and increases in IL-6 and TNF-α are attributed to detrimental tissue-related complications (20). Here we showed that both acetylcholine and nicotine were able to attenuate, significantly, both TNF-α and IL-6 activity, whilst neither tacrine nor the targeting antibodies were effective. In addition, a small decrease in production of the anti-inflammatory cytokine IL-10 was noticed, in the presence of mCRP, but this tended to return to basal levels in the presence of acetylcholine or 3H12 antibodies. A reduction in anti-inflammatory cytokines elicited by mCRP could potentially alter the vascular micro-environment leading to enhanced inflammation and hence molecules that could block this effect systemically could have some therapeutic interest (21). Il-1β was also tested and substantially increased in the presence of mCRP, however, neither the antibodies nor the small molecules used showed any significant inhibition of cytokine expression (data not shown).

Previous work has demonstrated mCRP induced production of IL-6 and TNF-α in U937 macrophages via Fc-gamma receptor-associated signaling and that co-incubation with oxidized LDL antagonized this inflammatory response (3). This pair of cytokines are linked in systemic inflammation/acute and chronic infection and are associated with increased risk of atherosclerosis and thrombosis and therefore blocking their production with a novel inhibitor such as acetylcholine could protect highly at risk individuals (22).

To confirm that macrophage cell signaling was perturbed, we carried out Western blotting experiments. Previously, Li et al. (23) showed that EC stimulation with mCRP induced MAP kinase signaling and p-38, NF-κB, associated with increase in IL-6 cytokine expression. Only one published study investigating the effects of mCRP on macrophage signaling was carried out previously by Eisenhardt et al. (24) who performed proteomic analysis on THP-1 macrophages, but did not identify any critical signaling intermediates associated with pro-inflammatory gene expression. We pre-incubated macrophages with acetylcholine or nicotine (since they produced a strong inhibitory inflammatory response in the presence of mCRP) and observed a reduction in p-ERK1/2 by both, and NF-κB phosphorylation in the presence of both acetylcholine and nicotine (p-JNK was weakly inhibited whilst AKT/p-p38 were not affected—AKT not shown). mCRP also caused nuclear translocation of NF-κB (by immunofluorescent analysis, for which the data are derived from only duplicate experiments)-a process associated with phosphorylation and degradation of IƙBα normally allowing translocation of NFkB into the nucleus where it regulates gene transcription).

The phosphorylation of NF-κB by acetylcholine when applied alone is difficult to explain but this was previously reported following incubation with a bronchial epithelial cell line, and linked to increased IL-8 production, although the mechanism responsible for this is not clearly understood (25). Oenema et al. showed stimulation of IƙB in smooth muscle cells through the muscarinic receptors indicating a possible signaling mechanism for this surprising finding (26).

Previous work has shown that both p-38 and NF-κB are required for IL-6/TNF-α processing in multiple cell types (27), and hence our work provides an indication that, particularly, acetylcholine, may block mCRP binding and signaling pathways associated with its powerful pro-inflammatory action.

We assessed whether there was a direct interaction between mCRP/nCRP and small molecules to indicate if direct binding possibly leading to structural modification, may be responsible for imparting biological inhibition. Dot blots performed on nitrocellulose bound with specific mCRP antibodies showed no ability of the small molecules to block binding directly to the antibody, nor to inhibit native CRP dissociation in the presence of urea. Similarly SPR could not show any direct interaction apart from the antibody (which we used as a positive control), thus indicating the interaction of these substances with mCRP may be at the membrane-phosphotidylcholine binding site rather than specific binding to the CRP which should be the subject of further investigation.

To further investigate the effects on macrophage activation and the relationship with EC adhesion, we conducted the Cytoselect monocyte-EC adhesion assay. Previous work has shown an important role for mCRP in stimulation of neutrophil attachment to human coronary artery EC [HCAEC; (28)], whilst Khreiss et al. (29), showed mCRP-induced HCAEC through enhanced MCP-1 and IL-8 secretion with concomitant phospho-p-38 expression, although there is no specific literature describing the link between EC and macrophages. Recently, mCRP was shown to activate angiogenesis and trigger F3 gene transcription, upregulating tissue factor signaling (30). Here we show that mCRP induced EC-monocyte adhesion and this was notably inhibited in the presence of both nicotine and acetylcholine (although not tacrine), and also, similarly using our two targeting antibodies, although these trends were non-significant. Inflammation and cell “stickiness” linked to mCRP are known to encourage monocyte attachment to the vascular cell wall for example at the early stages of atherosclerosis (31), and later as a precipitant of thrombosis with platelet aggregation involvement (32).

Regarding platelet aggregation, mCRP at 100 μg/ml was previously shown to cause CD62-platelet aggregation and adhesion to fibrinogen (33). Using our standardized thrombotic assay, we showed that mCRP (100 μg/ml) significantly induced thrombosis within 2 min of application (~39%). In the presence of small molecules/antibodies, whilst the 3H12 was ineffective, the 8C10 antibody and all three small molecules significantly attenuated platelet aggregation, whilst the positive control ADP was not blocked. Mollins et al. (32), partially explained the mechanism of mCRP action through surface P-selectin activation, CD63 exposure, and glycoprotein IIb-IIIa activation. Although we are not sure of the characteristics of our small molecules, the thrombotic pathway appears to be driven at least partially through p-38 activation and at least acetylcholine was able to block this pathway hence this could help to explain our findings. Regarding the anti-mCRP antibodies, the 8C10 binds to the N-terminal part of mCRP through aa 22–45 thereby covering the cholesterol binding site and probably explaining a mechanism for prevention of mCRP from entering lipid rafts (2). This may explain its greater effectiveness when compared with the 3H12 antibody which binds the C-terminal octapeptide aa 198–206. Since this epitope becomes hidden after mCRP enters a lipid zone e.g., on the surface of a cell membrane or lipid rafts of platelets, mCRP may have bound to the platelets prior to an effective influence of the antibodies or small molecules since there was no pre-incubation phase in this experiment.

We confirm here that small molecules like acetylcholine and nicotine could potentially be developed or optimized as protectors in cardiovascular and other inflammatory debilitating conditions. Nonetheless acetylcholine administration may have considerable severe side effects when administered systemically, (e.g., inhibition of other normal CNS functions by blocking serotonin function) leading to enhanced anxiety and depression (34). In addition, since the normal half-life of acetylcholine in the blood is 1–2 min, treatment requiring prolonged action systemically would require additional anticholinesterase therapy, and in fact, tacrine, is an example of a drug previously tested for management of Alzheimer's within the USA (35).

It is worthy of note that whilst several years ago, there was some controversy over the existence of mCRP *in vivo*, and identification of the active rmCRP; an intermediate form produced on contact of the native protein with cell membranes and liposomes (36, 37). More recent work from Thiele et al. (4) and others, demonstrated manipulation of CRP using a specific phosphocholine inhibitor (1-6-bis(phosphocholine)-hexane), *in vivo*. They showed the existence of mCRP in tissue and pharmacologically successfully blocked this dissociation directly at the cell surface, thereby validating our studies here and indicating a possible novel therapeutic strategy to abrogate inflammatory disease.

In conclusion, orphan, off target molecules such as acetylcholine or more specific small molecules, of similar structure may have potential for blocking the pro-inflammatory effects of CRP.

## Author contributions

MS, MD, W-HF, RA, XZ, and GM designed the project, experiments, managed the work, and drafted the script. RI and BG conducted the ELISA and inflammation assays. DL and RI conducted the Western blotting. GF did the adhesion assay. YZ and NB performed and managed the platelet aggregation assay. VC organized all the small molecule studies and NP carried out the SPR.

### Conflict of interest statement

The authors declare that the research was conducted in the absence of any commercial or financial relationships that could be construed as a potential conflict of interest.

## Supplementary material

Supplementary Figure 1 Shows trypan blue cytotoxicity screening and no significant cell death or loss of viability after 24 h in the presence of the following concentrations of the small molecules /nicotinic acid receptor inhibitors used within this study (acetylcholine-0–500 μM; nicotine-0–150 ng/ml; methyllycaconitine citrate-0–200 μM). Each experiment was performed three times in triplicate and a representative example is shown here.

Supplementary Figure 2 Results for a second donor for the platelet aggregation assay showing a similar pattern of results for mCRP and the effects of the small molecules on aggregation. Anti-mCRP antibody 8C10 blocked effectively the aggregation of platelets by mCRP whilst 3H12 only partially abrogated the effects (*P* < 0.05). Acetylcholine (10 μM), nicotine (0.93 μM), and tacrine (5 μM), all significantly inhibited mCRP-induced platelet aggregation. Results are presented as the mean ± SD from a representative example (different donor) of three independent experiments. ^*^*P* ≤ 0.05; ^**^*P* < 0.01; ^***^*P* < 0.001 using one-way ANOVA with Bonferroni post-test analysis.

## References

[1] Eisenhardt SU Habersberger J Peter K. Monomeric C-reactive protein generation on activated platelets: the missing link between inflammation and atherothrombotic risk. Trends Cardiovasc Med. (2009) 19:232–7. 10.1016/j.tcm.2010.02.002. 20382347

[2] Ji SR Ma L Bai CJ Shi JM Li HY Potempa LA. Monomeric C-reactive protein activates endothelial cells via interaction with lipid raft microdomains. FASEB J. (2009) 23:1806–16. 10.1096/fj.08-116962 19136614

[3] Trial J Potempa L Entman ML. The role of C-reactive protein in innate and acquired inflammation: new perspectives. Inflamm Cell Signal. (2016) 3:E1409. 10.14800/ics.1409 27738646 PMC5058362

[4] Thiele JR Habersberger J Braig D Schmidt Y Goerendt K Maurer V. Dissociation of pentameric to monomeric C-reactive protein localizes and aggravates inflammation: *in vivo* proof of a powerful proinflammatory mechanism and a new anti-inflammatory strategy. Circulation (2014) 130:35–50. 10.1161/CIRCULATIONAHA.113.007124 24982116

[5] Slevin M and Krupinski J. A role for monomeric C-reactive protein in regulation of angiogenesis, endothelial cell inflammation and thrombus formation in cardiovascular/cerebrovascular disease? Histol Histopathol. (2009) 24:1473–8. 10.14670/HH-24.1473 19760596

[6] McCann SK Cramond F Macleod MR Sena ES. Systematic review and meta-analysis of the efficacy of interleukin-1 receptor antagonist in animal models of stroke: an update. Transl Stroke Res. (2016) 7:395–406. 10.1007/s12975-016-0489-z 27526101 PMC5014900

[7] Pulicherla KK Verma MK. Targeting therapeutics across the blood brain barrier (BBB), prerequisite towards thrombolytic therapy for cerebrovascular disorders-an overview and advancements. AAPS PharmSciTech. (2015) 16:223–33. 10.1208/s12249-015-0287-z 25613561 PMC4370956

[8] Shytle RD Mori T Townsend K Vendrame M Sun N Zeng J. Cholinergic modulation of microglial activation by alpha 7 nicotinic receptors. J Neurochem. (2004) 89:337–43. 10.1046/j.1471-4159.2004.02347.x 15056277

[9] Park HJ Lee PH Ahn YW Choi YJ Lee G Lee DY. Neuroprotective effect of nicotine on dopaminergic neurons by anti-inflammatory action. Eur J Neurosci. (2007) 26:79–89. 10.1111/j.1460-9568.2007.05636.x 17581257

[10] Ezoulin MJ Liu Z Dutertre-Catella H Wu G Dong CZ Heymans F. A new acetylcholinesterase inhibitor with anti-PAF activity modulates oxidative stress and pro-inflammatory mediators release in stimulated RAW 264.7 macrophage cells. Comparison with tacrine. Int Immunopharmacol. (2007) 7:1685–94. 10.1016/j.intimp.2007.08.023 17996678

[11] Diehl EE Haines GK III Radosevich JA Potempa LA. Immunohistochemical localization of modified C-reactive protein antigen in normal vascular tissue. Am J Med Sci. (2000) 319:79–83. 10.1097/00000441-200002000-00002 10698090

[12] Slevin M Matou-Nasri S Turu M Luque A Rovira N Badimon L. Modified C-reactive protein is expressed by stroke neovessels and is a potent activator of angiogenesis *in vitro*. Brain Pathol. (2010) 20:151–65. 10.1111/j.1750-3639.2008.00256.x 19170684 PMC8094831

[13] Kapitsinou PP Sano H Michael M Kobayashi H Davidoff O Bian A. Endothelial HIF-2 mediates protection and recovery from ischemic kidney injury. J Clin Invest. (2014) 124:2396. 10.1172/JCI69073 24789906 PMC4092875

[14] Fujita M Takada YK Izumiya Y Takada Y. The binding of monomeric C-reactive protein (mCRP) to Integrins αvβ3 and α4β1 is related to its pro-inflammatory action. PLoS ONE (2014) 9:e93738. 10.1371/journal.pone.0093738 24695572 PMC3973595

[15] de la Torre R Peña E Vilahur G Slevin M Badimon L. Monomerization of C-reactive protein requires glycoprotein IIb-IIIa activation: pentraxins and platelet deposition. J Thromb Haemost. (2013) 11:2048–58. 10.1111/jth.12415 24119011

[16] Slevin M Matou S Zeinolabediny Y Corpas R Weston R Liu D. Monomeric C-reactive protein-a key molecule driving development of Alzheimer's disease associated with brain ischaemia? Sci Rep. (2015) 3:13281. 10.1038/srep13281 26335098 PMC4558604

[17] Chirco KR Whitmore SS Wang K Potempa LA Halder JA Stone EM. Monomeric C-reactive protein and inflammation in age-related macular degeneration. J Pathol. (2016) 240:173–83. 10.1002/path.4766 27376713 PMC5527328

[18] Cuello F Shankar-Hari M Mayr U Yin X Marshall M Suna G. Redox state of pentraxin 3 as a novel biomarker for resolution of inflammation and survival in sepsis. Mol Cell Proteomics (2014) 13:2545–57. 10.1074/mcp.M114.039446 24958171 PMC4188985

[19] Nazarov PG Krylova IB Evdokimova NR Nezhinskaya GI Butygov AA. C-reactive protein a pentraxin with anti-acetylcholine activity. Life Sci. (2007) 80:2337–41. 10.1016/j.lfs.2007.04.031 17531271

[20] Krayem I Bazzi S Karam M. The combination of CRP isoforms with oxLDL decreases TNF-α and IL-6 release by U937-derived macrophages. Biomed Rep. (2017) 3:272–6. 10.3892/br.2017.949 28808571 PMC5543421

[21] Singh U Devaraj S Dasu MR Ciobanu D Reusch J Jialal I. C-reactive protein decreases interleukin-10 secretion in activated human monocyte-derived macrophages via inhibition of cyclic AMP production. Aterioscler Thromb Vasc Biol. (2006) 26:2469–75. 10.1161/01.ATV.0000241572.05292.fb 16917108

[22] Ridker PM Luscher TF. Anti-inflammatory therapies for cardiovascular disease. Eur Heart J. (2014) 35:1782–91. 10.1093/eurheartj/ehu203 24864079 PMC4155455

[23] Li HY Wang J Wu YX Zhang L Liu ZP Filep JG. Topological localization of monomeric C-reactive protein determines proinflammatory endothelial cell responses. J Biol Chem. (2014) 16:289:14283–90. 10.1074/jbc.M114.555318 24711458 PMC4022894

[24] Eisenhardt SU Habersberger J Oliva K Lancaster GI Ayhan M Woollard KJ. A proteomic analysis of C-reactive protein stimulated THP-1 monocytes. Proteome Sci. (2011) 9:1. 10.1186/1477-5956-9-1 21219634 PMC3023727

[25] Profita M Bonanno A Siena L Ferraro M Montalbano AM Pompeo F. Acetylcholine mediates the release of IL-8 in human bronchial epithelial cells by a NFkB/ERK-dependent mechanism. Eur J Pharmacol. (2008) 582:145–53. 10.1016/j.ejphar.2007.12.029 18242599

[26] Oenema TA Kolahian S Nanninga JE Rieks D Hiemstra PS Zuyderduyn S. Pro-inflammatory mechanisms of muscarinic receptor stimulation in airway smooth muscle. Respir Res. (2010) 11:130. 10.1186/1465-9921-11-130 20875145 PMC2955662

[27] Craig R Larkin A Mingo AM Thuerauf DJ Andrews C McDonough PM. p38 MAPK and NF-κB Collaborate to Induce Interleukin-6 Gene Expression and Release: evidence for a cytoprotective autocrine signalling pathway in a cardiac monocyte model system. J Biol Chem. (2000) 275:23814–24. 10.1074/jbc.M909695199 10781614

[28] Zouki C Haas B Chan JS Potempa LA Filep JG. Loss of pentameric symmetry of C-reactive protein is associated with promotion of neutrophil-endothelial cell adhesion. J Immunol. (2001) 167:5355–61. 10.4049/jimmunol.167.9.5355 11673552

[29] Khreiss T József L Potempa LA Filep JG. Loss of pentameric symmetry in C-reactive protein induces interleukin-8 secretion through peroxynitrite signaling in human neutrophils. Circ Res. (2005) 97:690–7. 10.1161/01.RES.0000183881.11739.CB 16123332

[30] Peña E de la Torre R Arderiu G Slevin M Badimon L. mCRP triggers angiogenesis by inducing F3 transcription and TF signalling in microvascular endothelial cells. Thromb Haemost. (2017) 117:357–70. 10.1160/TH16-07-0524 27808345

[31] Arakawa M Mita T Azuma K Ebato C Goto H Nomiyama T. Inhibition of monocyte adhesion to endothelial cells and attenuation of atherosclerotic lesion by a glucagon-like peptide-1 receptor agonist, exendin-4. Diabetes (2010) 59:1030–7. 10.2337/db09-1694 20068138 PMC2844811

[32] Molins B Peña E de la Torre R Badimon L. Monomeric C-reactive protein is prothrombotic and dissociates from circulating pentameric C-reactive protein on adhered activated platelets under flow. Cardiovasc Res. (2011) 1:92:328–37. 10.1093/cvr/cvr226 21859817

[33] Boncler M Rywaniak J Sicinska P Watala C. Effectiveness of modified C-reactive protein in the modulation of platelet function under different experimental conditions. Blood Coagul Fibrinoly. (2011) 22:301–9. 10.1097/MBC.0b013e3283451308 21372690

[34] Higley MJ Picciotto MR. Neuromodulation by acetylcholine: examples from schizophrenia and depression. Curr Opin Neurobiol. (2014) 29:88–95. 10.1016/j.conb.2014.06.004 24983212 PMC4268065

[35] Nair VP Hunter JM Anticholinesterases and anticholinergic drugs. Continuing Educ Anaesthesia Crit Care Pain (2004) 4:164–8. 10.1093/bjaceaccp/mkh045

[36] Ji SR Wu Y Zhu L Potempa LA Sheng FL Lu W. Cell membranes and liposomes dissociate C-reactive protein (CRP) to form a new, biologically active structural intermediate: mCRP(m). FASEB J. (2007) 21:284–94. 10.1096/fj.06-6722com 17116742

[37] Li HY Wang J Meng F Jia ZK Su Y Bai QF. An intrinsically disordered motif mediates diverse actions of monomeric c-reactive protein. J Biol Chem. (2016) 291:8795–804. 10.1074/jbc.M115.695023 26907682 PMC4861447

