# Supplementary figures and images for: Acetylcholine Inhibits Monomeric C-Reactive Protein Induced Inflammation, Endothelial Cell Adhesion, and Platelet Aggregation; A Potential Therapeutic?
Source: Front Immunol. 2018 Sep 26;9:2124. doi: 10.3389/fimmu.2018.02124 (PMC6168760; doi:10.3389/fimmu.2018.02124)

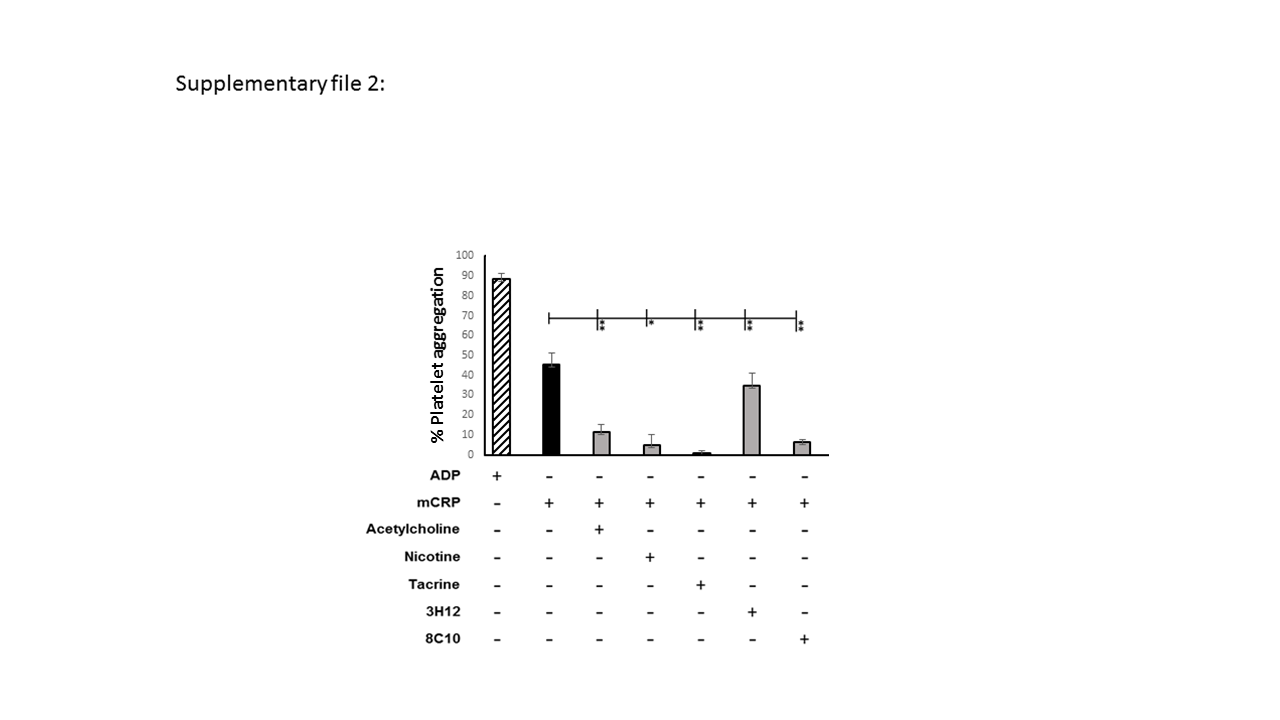

Supplement: Supplementary Figure 1 — Shows trypan blue cytotoxicity screening and no significant cell death or loss of viability after 24 h in the presence of the following concentrations of the small molecules /nicotinic acid receptor inhibitors used within this study (acetylcholine-0–500 μM; nicotine-0–150 ng/ml; methyllycaconitine citrate-0–200 μM). Each experiment was performed three times in triplicate and a representative example is shown here. [file Image_1.TIF]
